# Patient risk profiles and practice variation in nonadherence to antidepressants, antihypertensives and oral hypoglycemics

**DOI:** 10.1186/1472-6963-7-51

**Published:** 2007-04-10

**Authors:** Liset van Dijk, Eibert R Heerdink, Dinesh Somai, Sandra van Dulmen, Emmy M Sluijs, Denise T de Ridder, Anna MGF Griens, Jozien M Bensing

**Affiliations:** 1 NIVEL, Netherlands Institute for Health Services Research, P.O. Box 1568, 3500 BN Utrecht, The Netherlands; 2 Utrecht University; Faculty of Pharmaceutical Sciences, Department of Pharmacoepidemiology and Pharmacotherapy, Utrecht Institute for Pharmaceutical Sciences (UIPS), Utrecht, The Netherlands; 3 Utrecht University, Department of Health Psychology, PO Box 80140, 3508 TC Utrecht, The Netherlands; 4 Foundation for Pharmaceutical Statistics, The Hague, The Netherlands

## Abstract

**Background:**

Many patients experience difficulties in following treatment recommendations. This study's objective is to identify nonadherence risk profiles regarding medication (antidepressants, antihypertensives, and oral hypoglycemics) from a combination of patients' socio-demographic characteristics, morbidity presented within general practice and medication characteristics. An additional objective is to explore differences in nonadherence among patients from different general practices.

**Methods:**

Data were obtained by linkage of a Dutch general practice registration database to a dispensing registration database from the year 2001. Subjects included in the analyses were users of antidepressants (n = 4,877), antihypertensives (n = 14,219), or oral hypoglycemics (n = 2,428) and their GPs. Outcome variables were: 1) early dropout i.e., a maximum of two prescriptions and 2) refill nonadherence (in patients with 3+ prescriptions); refill adherence < 80% was considered as nonadherence. Multilevel modeling was used for analyses.

**Results:**

Both early dropout and refill nonadherence were highest for antidepressants, followed by antihypertensives. Risk factors appeared medication specific and included: 1) non-western immigrants being more vulnerable for nonadherence to antihypertensives and antidepressants; 2) type of medication influencing nonadherence in both antihypertensives and antidepressants, 3) GP consultations contributing positively to adherence to antihypertensives and 4) somatic co-morbidity influencing adherence to antidepressants negatively. There was a considerable range between general practices in the proportion of patients who were nonadherent.

**Conclusion:**

No clear risk profiles for nonadherence could be constructed. Characteristics that are correlated with nonadherence vary across different types of medication. Moreover, both patient and prescriber influence adherence. Especially non-western immigrants need more attention with regard to nonadherence, for example by better monitoring or communication. Since it is not clear which prescriber characteristics influence adherence levels of their patients, there is need for further research into the role of the prescriber.

## Background

Adherence to medication can be defined as the extent to which patients follow the instructions they are given for prescribed treatments [1]. Many patients experience difficulties in following treatment recommendations [2]. As a result levels of adherence are often far from optimal, especially in patients with chronic diseases. Nonadherence is supposed to have a negative impact on patient outcomes in terms of medical and psychosocial complications of disease, reduction in patients' quality of life and waste of health care resources [2,3].

Adherence rates have hardly changed over the last decades [4]. In medical practice, patients are usually reluctant to talk about adherence. Therefore, it will be helpful to identify nonadherent patients by their risk profiles. Since adherence problems are influenced by many factors, risk profiles should include patient-related, disease and treatment characteristics as well as attributes of the healthcare system [2,5,6]. Patient-related characteristics that, for example, have been correlated with nonadherence are socio-economic status, etnicity and social support [2].

Although many studies have been performed on determinants of nonadherence, no clear picture emerges of the "nonadherent" patient [3]. The WHO-report suggested that factors associated with nonadherence differ by type of disease [2]. However, there is hardly any research that uses comparable data on different diseases [7]. So far, differences can as much be the result of using different outcome measures, different correlates, different patients or the fact that, for example, adherence to antihypertensives is influenced by other factors than antidepressants. A reason why patient risk profiles may be hard to construct, is that adherence is not only determined by patient characteristics but also by variation between prescribers in adherence levels of their patients. While it is obvious that patients differ in level of adherence, it is not clear to what extent prescribers vary in the proportion of nonadherent patients. This is because, up to now, patients have been the main focus of research.

This study's objective is to identify risk profiles regarding nonadherence from a combination of patients' socio-demographic characteristics, morbidity presented within general practice and medication characteristics. For this study we selected medication use for three chronic diseases:

1) depression

2) hypertension

3) type 2 diabetes.

These diseases are known for their lifelong or long-term medication need and identified as high risk areas regarding adherence [2]. For all three diseases the same source population as well as the same outcome measures will be used. An additional objective is to explore differences in adherence among patients from different general practices.

## Methods

### Study setting

Data for this study were obtained by linkage of routine registration data collected in general practice to a dispensing registration database. In the Netherlands, every individual is listed in a general practice. In 2001, the Netherlands had 7,763 GPs [8]. GP data were used from the Second Dutch National Survey of General Practice (DNSGP-2). This representative survey of 104 Dutch general practices (195 GPs) and their 385,461 patients was performed by NIVEL (Netherlands Institute for Health Services Research) in cooperation with RIVM (National Institute for Public Health) in 2001 [9]. Registration data were collected on all medical consultations, prescriptions, and referrals. Furthermore, 76.5% of the patients responded to a census with questions on demographic data, such as age, gender, health insurance, educational level, and perceived health. Most census data were collected in 2000. Pharmacy dispensing data were collected by the Foundation for Pharmaceutical Statistics (SFK). The SFK network represents 86.5% of all public pharmacies in the Netherlands. Medication histories of Dutch pharmacies are virtually complete because almost all patients fill their prescription at a single pharmacy and prescriptions written by GPs and medical specialists are included. Data from both sources were linked by researchers from Utrecht University. In short, 110,102 patients from 83 general practices participating in DNSGP-2 were identified in 112 pharmacies who delivered data to SFK. Linking was based on the patient's gender, year of birth, postal code and prescription characteristics. Prescription characteristics were used in the linking process because the other three linking keys did not provide unique matches for all patients. Since both DNSGP-2 and SFK included data on patients' medication it was possible to compare medication profiles (type of medication, date of prescribing) as far as prescribed by the GP. The linking procedure is described and discussed in more detail elsewhere [10].

The study was carried out according to Dutch legislation on privacy. The privacy regulation of the study was approved by the Dutch Data Protection Authority. According to Dutch legislation, obtaining informed consent is not obligatory for observational studies.

### Subjects

Subject to the study were patients who used antidepressants, antihypertensives or oral hypoglycemics in 2001 for whom a successful linking was achieved and who had no missing data on the included variables. Data were available for 7,365 users of antidepressants, 20,004 users of antihypertensives and 3,548 users of oral hypoglycemics. In total, 2,488 users of antidepressants had missing data on sociodemographic variables and morbidity (33.8%); the same was true for 5,785 users of antihypertensives (28.9%) and 1,120 users of oral hypoglycemics (31.6%). Patients with missing data did not differ from the included patients on nonadherence measures.

### Measures

#### Nonadherence

Two measures for nonadherence were calculated: 1) early dropout and 2) refill of prescriptions ('refill nonadherence'). Early dropout was defined as a maximum of two prescriptions in 2001 and no preceding prescriptions in the last half of 2000 or subsequent prescriptions in the first half of 2002. Drop-out rates for oral hypoglycemics were low (n = 82). Therefore, this measure was only calculated for antihypertensives and antidepressants.

Regarding refill nonadherence we adopted a procedure developed by the Utrecht Institute for Pharmaceutical Sciences (UIPS) to calculate nonadherence among chronic users of medication. To calculate refill nonadherence we first defined the date of the first prescription in 2001 as well as the end date of the last prescription initiated in 2001. This end date usually was in 2002. Once the start and end date were determined, we calculated the number of days for which dosages were dispensed between the start and the end date divided by the total number of days between the start and end date. Following other studies, a patient with compliance below 80% was considered to be nonadherent [7,11]. We calculated this measure for patients with chronic medication, i.e. those who had at least three prescriptions in 2001. Of course, we preferred to use cut-off points for which evidence has shown its relevance for outcomes. However, strong evidence for the best cut-off point per disease is not yet available. Therefore, we took the same cut-off point for all three types of medication: 80%. The reason to choose for 80% is twofold. First, we expected risk profiles to be more clear using this cut-off point, compared to, for example, a cut-off point of 90%- 95% or a continuous measure. Second, a pragmatic reason was that if thresholds < 80% were used, the absolute number of patients in the nonadherent groups was low, especially for oral hypoglycemics.

#### Socio-demographic characteristics

Gender and age were included. As indicators for social status we included educational level (0=precollege education; 1 = college/university), type of health insurance (0 = public; 1 = private) and employment status (0 = not employed or in school; 1 = employed or in school). Furthermore, first and second generation immigrants from non-western countries were compared to the combined population of western immigrants and the indigenous Dutch population (0 = western; 1 = non-western). Finally, we included a variable that reflects whether or not the patient lives together (0 = living alone; 1 = living with spouse and/or children).

#### Medication

All prescriptions in the database were coded using the Anatomical Therapeutical Chemical (ATC) coding system [12]. Antidepressants (ATC-code N06A) were divided into three categories: TCAs (N06AA), SSRIs (N06AB) and other antidepressants (N06AF/N06AX). The following antihypertensives were distinguished: diuretics (C03), beta-blockers (C07), ACE-inhibitors/angiotensin II receptor antagonists (C09) and other (C02/C08). For oral hypoglycemics no such distinction was made; these drugs closely resemble each other when it comes to adverse reactions, effectiveness etc (oral information EH). We tested whether adherence to biguanides and sulfonylureas indeed did not differ. We found no significant differences. Patients who used insulin were not included in the analysis. As a proxy for complex medication regime the sum of different full ATC-codes was used.

#### Morbidity presented in general practice

The contact registration in the DNSGP-2 database provided information on the diagnoses for which the patient consulted the GP. Every contact was coded according to the International Classification for Primary Care (ICPC) [13]. We assessed whether the patient visited the GP for the main indications for respectively antidepressants, antihypertensives, and oral hypoglycemics: depression (P03/P76) or anxiety (P01/P74), hypertension (ICPC: K85, K86, K87), and diabetes mellitus (T90). Moreover, we assessed whether patients visited their GP during the year of registration for related complaints such as other cardiovascular diseases (K70–K90, excluding hypertension), and hypercholesterolemia and, for antidepressants, other psychological diagnoses in the ICPC-chapter P. The total number of other chronic diseases for which the patient contacted the GP in 2001 was also assessed. The definition of chronic diseases was adopted from earlier studies [14]. The disease under study and related diseases were excluded from the definition; they were already covered by the variables described above. While the database included information on prescriptions by medical specialists, information on consultation of medical specialists is lacking in the database. We also included information on self-perceived health, which was asked in the patient census (0 = poor to moderate; 1 = good to excellent). Finally, the total number of contacts the patient had with the GP in 2001 was calculated.

### Analysis

Chi-square tests and student's t-test were used to analyze differences in basic characteristics between patients who were adherent and patients who were not. These bivariate analyses did not take into account the clustered nature of our data. We did use the nested structure of the data – our patients were sampled from general practices – in the multivariate analyses. We fitted a binomial 2^nd ^order multilevel model with two levels (practice and patient) for each of the five dependent variables of interest (two times early dropout, three times refill nonadherence). All independent variables (sociodemographics, use of medication and health and morbidity) were included in the same multilevel model, so all independent variables in the multilevel analyses were adjusted for each other.

As we stated before, the 80% cut-off point for nonadherence is arbitrary [7]. Therefore, we also estimated the same models for refill adherence using 70% (despite low numbers of patients for oral hypoglycemics) and 90% as cut-off points. Moreover, we used a linear model to estimate correlates between the independent variables and Nonadherence measured as a continous variable. The results of these analyses are not presented in tables, but they are discussed related to the results of the analyses using the 80% cut-off point.

Multilevel analysis was also used to calculate the probability that a patient is refill nonadherent at the practice level. We expressed this as the proportion of nonadherent patients in the practice. We used an empty model only including the dependent variables and the two levels (patient and practice). In that way the "entrance" probability of a patient to be nonadherent in a certain practice is known. Such model also allows to calculate the 95%-confidence interval for this probability. This interval shows the range between the minimum and maximum probability to be nonadherent at the practice level. The model was fitted for each drug therapy.

All data were analyzed with SPSS version 11.0 and 'multi-level models for windows' (MLwin).

## Results

### Antidepressants

In total 4,877 patients were eligible for analyses of whom 928 patients received one or two prescriptions in 2001. 556 of them could be identified as an early dropout because they received no prescription for antidepressants the half year before and after 2001. This implies that 11.4% of the 4,877 included patients were early dropouts. Women were less likely and non-western immigrants were more likely to be an early dropout (Table 1/Additional file 1). Users of TCAs were overrepresented among the early dropouts. Early dropouts were more likely to consult their GP for depression and anxiety than other patients; the opposite holds for neurasthenia and other psychological problems. On average, early dropouts presented more other chronic diseases to their GP than other users.

**Table 1:** Nonadherence in antidepressant use: results of the binomial multilevel analyses on early dropouts and continuers and on refill nonadherence

	**Early dropout (0 = continuer; 1 = early dropout)**		**Refill adherence (0 = adherent > 80%; 1 = nonadherent)**	
	Oddsratio^a)^	95% CI	Oddsratio^a)^	95% CI
	
**Socio-demographic characteristics**				
-age (mean; SD)	1.00	[0.99–1.01]	1.00	[1.00–1.00]
-% woman	0.78*	[0.63–0.95]	1.00	[0.84–1.19]
-% college/university	1.05	[0.80–1.36]	0.86	[0.69–1.09]
-% non-western	2.47*	[1.70–3.60]	2.59*	[1.75–3.82]
-% private insurance	1.06	[0.85–1.33]	0.95	[0.78–1.15]
-% living together	1.02	[0.80–1.29]	1.00	[0.82–1.21]
-% with job/study	1.26	[1.00–1.60]	0.88	[0.73–1.07]
				
**Use of medication**				
*Antidepressants*				
- % users of SSRIs	0.80	[0.59–1.08]	1.27*	[1.04–1.63]
- % users of TCAs	1.55*	[1.14–2.11]	0.76*	[0.58–0.99]
- % users of other antidepressants	reference		reference	
*Complex regime*				
number of other ATCs (mean;sd)	1.02	[1.00–1.05]	0.97*	[0.95–0.99]
				
**Health & morbidity in general practice**				
*Self-reported health*				
% excellent/good	1.20	[0.98–1.47]	1.17	[0.99–1.37]
*Diagnoses for which GP is consulted (% of patients)*				
Depression (P03/P76)	0.62*	[0.50–0.76]	0.91	[0.77–1.07]
Anxiety (P01/P74)	0.75*	[0.57–0.98]	1.15	[0.93–1.42]
Neurasthenia (P78)	2.01*	[1.34–3.04]	0.82	[0.51–1.31]
Other diagnoses in P-chapter (P01–P99)	1.18	[0.95–1.47]	1.17	[0.97–1.41]
*GP consultation for chronic diseases and overall contact*				
Number of other chronic complaints (mean, SD)	1.14*	[1.05–1.24]	1.18*	[1.09–1.27]
Number of contacts with GP (mean; SD)	0.99	[0.98–1.00]	0.99	[0.98–1.00]
Number of patients (N)	4,877		3,777	

* P < 0.05

a) odds ratio > 1: more likely to be an early dropout; odds ratio < 1: less likely to be an early dropout

In total, 3,777 patients received three or more prescriptions in 2001; for these patients refill adherence was calculated. Almost a quarter of them (24.6%) showed a refill rate < 80%. We found that non-western immigrants were more likely to be nonadherent (Table 1/Additional file 1). Refill nonadherence was highest for SSRIs and lowest for TCAs. A complex medication regime (i.e. more different types of medication) was moderately negatively associated with refill nonadherence. Patients were more likely to be nonadherent the more other chronic complaints they had. Patients who rate their health as good-excellent have lower compliance. These two last findings show that those who feel good, are less likely to take their medication regardless of their actual disease pattern. The analyses using other cut-off points (70%, 90%) and the analysis using refill adherence as a continuous dependent variable all showed identical significant effects for ethnicity and other chronic complaints. They did not yield any new significant relationship, except that both the analysis with the 70% cut-off point and the analysis with the continuous measure showed that patients who consulted their GP for anxiety were less often nonadherent.

### Antihypertensives

In total 2,109 patients received only one or two prescriptions in 2001; 813 of them could be identified as an early dropout because they had no prescriptions in the half year before and/or after 2001. Therefore, 5.7% of the 14,219 patients included in the analyses was considered to be an early dropout. Compared to continuers, early dropouts were more often younger, female, higher educated or had a non-western background (Table 2/Additional file 2). Users of diuretics were more likely to be an early dropout than patients who used any of the other antihypertensives. The chance that early dropouts consulted their GP for hypertension and related co-morbidity was lower than that of other users of antihypertensives. Moreover, early dropouts presented more other chronic complaints in general practice, but they used less different types of medication. Since early dropouts proved to be clearly younger than other patients, we also performed an analysis in which only patients > 40 years were included. The effects for this population proved to be weaker, but still were significant (results not in table).

**Table 2:** Nonadherence in antihypertensive use: results of the binomial multilevel analyses on early dropouts and continuers and on refill nonadherence

	**Early dropout (0 = continuer; 1 = early dropout)**		**Refill adherence (0 = adherent > 80%; 1 = nonadherent)**	
	Odds ratio^a)^	95% CI	Odds ratio^a)^	95% CI
	
**Socio-demographic characteristics**				
- age (mean; SD)	0.95*	[0.95–0.96]	1.00	[0.99–1.00]
- % woman	1.29*	[1.09–1.54]	1.08	[0.95–1.23]
- % college/university	1.34*	[1.06–1.69]	1.04	[0.85–1.27]
- % non-western	2.22*	[1.53–3.24]	1.44	[0.97–2.15]
- % private insurance	1.12	[0.93–1.35]	1.09	[0.95–1.25]
- % living together	0.94	[0.71–5.17]	0.88	[0.77–1.02]
- % with job/study	1.08	[0.88–1.32]	1.07	[0.89–1.27]
				
**Medication**				
*Antihypertensives*				
- % users of beta blockers	0.59*	[0.49–0.72]	0.50*	[0.44–0.57]
- % users of diuretics	ref.		reference	
- % users of ace-inhibitors/A2 antagonists	0.25*	[0.17–0.36]	0.37*	[0.31–0.45]
- % users of other antihypertensives	0.58*	[0.43–0.78]	0.30*	[0.23–0.38]
*Complex regime*				
number of other ATCs (mean;sd)	0.94*	[0.92–0.96]	0.97*	[0.96–0.99]
				
**Health & morbidity in general practice**				
*Self-reported health*				
% excellent/good	1.51*	[1.27–1.81]	0.93	[0.82–1.05]
*Diagnoses for which GP is consulted (% of patients)*				
Diabetes (T90)	0.46*	[0.33–0.65]	0.69*	[0.60–0.91]
Hypertension (K85–K87)	0.11*	[0.08–0.13]	0.77*	[0.64–0.78]
Other diagnoses in K-chapter (from K70–K99)	0.76*	[0.62–0.78]	1.08	[0.98–1.24]
Hypercholesterolemia (T93)	0.50*	[0.32–0.78]	0.88	[0.94–1.10]
*GP consultation for chronic diseases and overall contact*				
Number of other chronic complaints (mean, SD)	1.11*	[1.02–1.21]	1.04	[0.98–1.11]
Number of contacts with GP (mean; SD)	1.04*	[1.03–1.05]	1.02*	[1.01–1.03]
Total (%)				
Number of patients (N)	14,219		12,110	

* P < 0.05

a) odds ratio > 1: more likely to be an early dropout; odds ratio < 1: less likely to be an early dropout

Refill adherence was calculated for the 12,110 patients who received three or more prescriptions in 2001. Refill adherence < 80% was found for 11.6% (n = 1,405) of them. Patients who used beta blockers, ace-inhibitors/A2-antagonists and other antihypertensives (C02/C09) were less likely to be nonadherent than users of diuretics. Moreover, patients with a complex medication regime were less likely to be nonadherent to antihypertensive medication; the effect, however, is moderate. Finally, chronic users who consulted their GP for hypertension or diabetes were more likely to take their medication as prescribed. In addition, both the analysis using the 90% cut-off point and the analysis using refill adherence as a continuous dependent variable showed that non-western immigrants were less adherent and that patients who live together are more likely to be adherent.

### Oral hypoglycemics

Of the 2,428 patients eligible for analysis 90.4% (n = 2,194) had three or more prescriptions in 2001. Refill adherence < 80% was found for 6.9% (n = 152) of them. Nonadherent patients hardly differed from adherent patients with regard to patient characteristics, medication and GP consultation (Table 3). Only two differences were found in the multilevel analysis: patients who lived together were less likely to be nonadherent to diabetes medication compared to patients who lived alone. Diabetes patients who visited their GP for cardiovascular diseases were also less likely to be nonadherent (< 80%). Since only 152 patients who used hypoglycemics were nonadherent, we also estimated models with less variables. These analyses did not show different results. However, the analysis using 90% as a cut-off point for nonadherence showed some extra correlates. Patients who were older, had a paid job and a complex medication regime were more often nonadherent.

**Table 3:** Refill adherence to oral hypoglycemics: differences between adherent and nonadherent patients

	**Bivariate analyses**		**Multilevel logistic analysis (multivariate; 0 = adherent; 1 = nonadherent)**	
	Nonadherent patients	Adherent patients	Oddsratio^a)^	95% CI
	
**Socio-demographic characteristics**				
- age in years (mean; SD)	66.6 (13.4)	65.8 (11.9)	1.01	[0.99–1.03]
- % woman	59.2	52.9	1.22	[0.82–1.82]
- % college/university	6.6	8.4	0.85	[0.40–1.82]
- % non-western	9.9	7.1	1.49	[0.76–2.91]
- % private insurance	19.7	23.9	0.75	[0.47–1.22]
- % living together	63.8*	73.5	0.65*	[0.43–0.97]
- % with job/study	19.1	17.9	1.68	[0.94–3.00]
				
**Use of medication**				
*Complex regime*				
number of other ATCs (mean;sd)	8.4 (6.0)	7.5 (5.3)	1.04	[0.99–1.08]
				
**Health & morbidity in general practice**				
*Self-reported health*				
% excellent/good	36.2	44.0*	0.75	[0.51–1.11]
*Diagnoses for which GP is consulted (% of patients)*				
Diabetes (T90)	84.2	88.2	0.69	[0.41–1.17]
Hypertension (K85–K87)	28.9	32.7	0.77	[0.51–1.17]
Other diagnoses in K-chapter (from K70–K99)	21.1	25.8	0.60*	[0.37–0.98]
Hypercholesterolemia (T93)	11.2	11.9	0.96	[0.52–1.77]
*GP consultation for chronic diseases and overall contact*				
Number of other chronic complaints (mean, SD)	0.7 (0.9)	0.7 (1.0)	0.87	[0.70–1.07]
Number of contacts with GP (mean; SD)	12.7 (10.8)	12.3 (9.9)	1.01	[0.98–1.03]
Total (%)	6.9			
Number of patients (N)	152	2,042	2,194	

* P < 0.05

a) odds ratio > 1: more likely to be nonadherent; odds ratio < 1: less likely to be nonadherent

### Differences between practices

An additional objective of this study was to explore differences in adherence among patients from different general. This was only calculated for the refill adherence outcome measure since for early dropout the number of patients per practice was generally low, leaving too few practices for analyses. Table 4 shows that there was a considerable range between general practices in the proportion of patients who were nonadherent. Differences between general practices were largest for hypoglycemics. This was confirmed in a multilevel analysis in which we estimated the 95% -confidence interval (CI-95%) for the proportion of non-adherent patients. For antidepressants CI-95% was 65–83%, for hypertension 74–95% and for oral hypoglycemics 73–98%. Moreover, analysis including patient characteristics showed that this variation cannot be explained by differences in patient population.

**Table 4:** Percentage of patients who were adherent (refill adherence) per general practice^a) ^and correlations between drugs^b)^

Type of drug	Proportion of adherent patients	95% CI interval	Correlations between drugs (for practices > 10 patients)		N
			Antihyper- tensives	Antidepressants	

Hypoglycemics	90.7	73–98%	0.53*	0.39*	65
Antihypertensives	86.7	74–95%		0.24	72
Antidepressants	77.9	65–83%			69

* p < 0.01

a) estimated with a multilevel model (empty model)

b) estimated at the practice level

There is a positive correlation between the proportion of adherent patients between the three drug groups, which is statistically significant (P < 0.05) for the association between 1) antidepressants and hypoglycemics (r = 0.39) and 2) antihypertensives and hypoglycemics (r = 0.53). These findings suggest that high adherence rates are at least partly a characteristic of the general practice.

## Discussion

This study found generally lower nonadherence rates compared to previous research for each of the three diseases [2]. However, definitions of nonadherence varied strongly across previous studies. The reason why we found lower rates of nonadherence may be that that we focused on early dropout and refill adherence < 80%. Moreover, our pharmacy record data did not include a measure for primary nonadherence, where the patient does not redeem the prescription [15]. In the dataset that was the source for this study, 7.6% of all prescriptions were not redeemed [16]. In absolute numbers beta blockers and antidepressants were among the top of non-redeemed drugs. However, these drugs also have a high prescription volumes in the Netherlands [17].

### Correlates for serious nonadherence

One of the aims of our study was to find out whether risk profiles for nonadherence were comparable for patients who used drugs for the following three chronic diseases: depression (antidepressants), hypertension (antihypertensives) and type 2 diabetes (oral hypoglycemics). Our main conclusion is that no such common risk profile emerges even when using the same source population and measurements. For different diseases and its related medication risk profiles differ and, therefore, nonadherence should be studied and treated disease-specific.

For oral hypoglycemics few correlates for nonadherence were found. On the contrary, early dropout from antihypertensive use was correlated with many factors. Hence, the question is whether these are indeed risk factors. For example, younger and highly educated patients were more likely to stop using antihypertensives at an early stage. These patients may find other ways to lower their blood pressure, for example by losing weight or doing more exercises.

We did find some important correlates for nonadherence to antidepressants and refill nonadherence to antihypertensives. First of all, we found that non-western immigrants were more vulnerable for nonadherence to both antihypertensives and antidepressants. This can be related to their socio-economic status but also to a lack of understanding about their disease and its treatment since not all immigrants are able to communicate easily in Dutch or English. Both poor socio-economic status and poor understanding are found to be related to lack of adherence in antihypertensives in previous studies [2,18]. Our findings cannot be attributed to moving out of the country and, therefore, out of the registration databases because non-western immigrants in the Netherlands have low emigration rates. In 2001, the year of our study, only 1% of Dutch non-western immigrants left the countr [19]. Our study also showed that in a primary care setting, type of medication is associated with nonadherence to both antidepressants and antihypertensives. Early discontinuation was higher among TCA-users. This may be due to the fact that TCAs are more often prescribed for other diagnoses such as pain, and ensuresis (in children). Once patients took antidepressants more continuously, users of TCAs proved to be most adherent users of antidepressants. This may be attributed to the fact that TCAs are more often subject to discontinuation because of side effects as was shown in a meta-analysis that compared treatment discontinuation of SSRIs and TCAs [20,21]. Once patients overcome the first prescriptions and either do not have side effects or accept them they may be more inclined to take the medication. For antihypertensives users of ace-inhibitors or/A2 antagonists were most adherent and type of medication proved to be the strongest correlate for adherence to antihypertensives. Nonadherence was highest among users of diuretics, which is in line with an earlier study in the Netherlands [22]. Studies from other countries not always found higher nonadherence rates for diuretics [23]. Lower adherence to diuretics is, for example, attributed to adverse effects and easiness of taking medication.

GP consultation is important for adherence to antihypertensives. Users of antihypertensives who visited their GP for hypertension and/or diabetes had higher adherence rates than patients who only had repeat medications (either from the GP or the medical specialist). This stresses the importance of communication about disease and treatment. Communication is facilitated by face-to-face consultation.

Somatic co-morbidity is associated with adherence to antidepressants: patients with somatic co-morbidity were more often seriously nonadherent. Since we controlled for complex medication regime (number of different type of drugs within a year) this effect cannot be attributed to difficulties encountered in taking multiple medications.

### In sum

For each definition of nonadherence we found that the nonadherent patient population is hard to characterize by its sociodemographic characteristics, GP consultation, and medication related information on the patient, especially since correlates – partly – vary across diseases. A prescriber determine out characteristics such as sociodemographics ans GP consultation pattern rather easily. However, adherence is probably also influenced by patient characteristics that are less visible and more subtle, for example the patients' attitudes towards taking medication or the patients' trust in the health care system [2]. These characteristics are more difficult to detect and need more time and attention from prescribers.

### Differences between practices

We found that adherence rates vary across general practices, even though the number of general practices included in the analysis was low (n = 72). Previous studies found that clear instructions on the management of disease has a positive impact on patient adherence [24] as has a good relationship between prescriber and patient [25]. Communication styles are found to differ between doctors [26,27]. Future research should therefore further unravel what characteristics and mechanisms cause patients from one general practice to be more adherent compared to patients listed in another practice.

### Strengths and limitations of the study

This study used a population-based dataset with a large sample, that enabled a multilevel analysis. Moreover, we combined registration information with data from a patient census, providing us with more information on the patient than most regular registration databases. However, our database also has some limitations. Dispensing general practices were excluded from the database, while about 10% of the patients in the Netherlands is listed in such practice. Moreover, not all patients could be linked. This was mainly due to the available linking keys [10,28]. The way the data were linked caused that the database included more patients with chronic medication. As our study included drugs that should be taken chronically, such bias towards our study is expected to be limited. We also had to exclude almost 30% of the (linked) patients for whom we had dispensing data and consultation data because these patients did not fill out the census form with patient characteristics. However, these patients did not differ in adherence rates from the patients who were included in the study.

A main advantage of refill data is that adherence rates can be estimated without the patient being aware of it. It increases the accuracy of the estimates by eliminating any Hawthorne effect [29]. Moreover, we used the medication refill adherence measurement (MRA), which is, according to a study by Hess et al [30] the preferred measure of adherence when using administrative data. The use of administrative pharmacy data also has some disadvantages. The first problem is that it is not possible to assess time of dosing [31,32] and that the data do not absolutely reflect patients' drug use. Roter et al (1998) suggested that prescription refills reflect patients' intention to comply rather than their actual drug consumption, i.e. patients fill their prescriptions more readily than they consume their medicine [33]. However, some authors argue that patterns of ongoing prescription refilling probably provide the most accurate estimate of actual medication use in large populations [34] , to assess drug exposure retrospectively or when direct measurement of medication is not feasible [31]. A recent study on hypertension medication found that compliance measured by electronic monitoring revealed higher adherence rates compared to prescription refills [34]. The researchers argued that the reason for this finding may be that electronic monitoring systems make patients aware of taking medication and as such influence adherence.

The data in our study refer mainly to the year 2001, because patient characteristics and morbidity data in general practice were only collected for that year. A one-year period may seem short to study adherence patterns. However, for refill adherence we only included patients with at least three prescriptions, which – given the fact that repeat prescriptions in the Netherlands often are prescribed for a three month period – covers about the whole year. In fact, this definition of refill adherence refers to patients who are inclined to used their medication but – in case they are nonadherent – who fail to do so.

Another problem is that the data do not tell what the reason for discontinuation is. Doctors may as well decide to stop the medication rather than the patient. As such we could not distinguish between a gap due to lack of adherence and a gap due to medical decisions. Since we expected this problem to be larger in the first stage of medication use, we separately analyzed early dropout from refill adherence. If a patient has three prescriptions over a one-year course (which was the minimal number of prescriptions for us to calculate refill adherence) we expect that there is an intention to continue the treatment. Moreover, our results showed that patients who consult their GP for complaints related to their medication (hypertension, anxiety, diabetes) are more compliant, which may be an indication that GPs are not very much inclined to stop the medication. Still, part of the discontinuation may be due to medical decisions and therefore, estimates for nonadherence in our study will be biased and – in real – levels of adherence will be higher. This may be especially true for antidepressants because this medication is not always chronic. However, over 80% of antidepressant users with three or more prescriptions in 2001 also had an antidepressant prescription in 2002, indicating that for the majority of patients there is an intention to continue treatment. A final disadvantage of the use of dispensing data is that such data do not reflect primary nonadherence.

### Implications for clinical practice and future research

For health care professionals it is hard to recognize nonadherent patients by their socio-demographic background. Moreover, socio-demographics that are correlated with nonadherence vary across different types of drugs. There is an exception, though: patients' ethnic background. Non-western immigrants have lower levels of adherence. In order to get insight into motivations of these patients to be nonadherent it is important to check upon and discuss this issue in patient-prescriber consultations: did they not understand why taking their medication is important, did they not agree upon taking medication etc. Cultural aspects may influence the attitude towards taking medication, an aspect we did not include in our study [35]. In fact, regular monitoring of and discussion on nonadherence is important for all patients.

We argue that guidelines for prescribers should include information on levels of nonadherence to certain types of medication. For example, the Dutch GP guideline for hypertension recommends diuretics as first choice medication in hypertension treatment. Our analyses showed that in Dutch general practice adherence to diuretics is lower than that to any other antihypertensive. For patients who are suspected by the prescriber to be nonadherent it may be more rational to prescribe a beta-blocker or an ace-inhibitor. At least prescribers should be aware of the nonadherence problem with this type of medication and closely monitor medication use. Such monitoring should include questions on adverse effects and or experiences in taking the medication during follow-up consultations.

Most of the implications mentioned refer to the importance of communication between professionals and patients. The fact that prescribers vary when it comes to their patients' adherence levels might, at least partly, be explained by differences in communication styles between prescribers. Further research into these differences among prescribers should provide more insight into this issue.

## Conclusion

No clear risk profiles for nonadherence could be constructed. Characteristics that were correlated with nonadherence varied across different types of medication. Moreover, both patient and prescriber influence adherence. Especially non-western immigrants need more attention with regard to nonadherence, for example by better monitoring or communication. Better insight into the role of the prescriber in explaining variation in adherence to medication is needed.

## Competing interests

The author(s) declare that they have no competing interests.

## Authors' contributions

JB and LvD formulated the research question and designed the study. LvD and DS carried out the study. LvD drafted and revised the manuscript. EH, DS and AG prepared the data. EH and DS advised on the analyses. All authors critically reviewed draft versions of the manuscript. All authors read and approved the final manuscript.

## Pre-publication history

The pre-publication history for this paper can be accessed here:



## Supplementary Material

Additional file 1 Appendix 1: Bivariate analyses antidepressants. The table represents the results of the bivariate analyses for antidepressants. Click here for file

Additional file 2 Appendix 2: Bivariate analyses antihypertensives. The table represents the results of the bivariate analyses for antihypertensives. Click here for file
