# Supplementary material for: Patient risk profiles and practice variation in nonadherence to antidepressants, antihypertensives and oral hypoglycemics
Source: BMC Health Serv Res. 2007 Apr 10;7:51. doi: 10.1186/1472-6963-7-51 (PMC1855317; doi:10.1186/1472-6963-7-51)
Supplement: Additional file 1 — Appendix 1: Bivariate analyses antidepressants. The table represents the results of the bivariate analyses for antidepressants. [file 1472-6963-7-51-S1.doc]

# Appendix 1: Bivariate analyses antidepressants

**Bivariate analyses on differences between early dropouts and continuers and on differences between seriously nonadherent patients (refill adherence)**

|  | **Early dropout** | | **Refill adherence** | |
| --- | --- | --- | --- | --- |
|  | Early dropout | Continuer | Nonadherent | Adherent |
| Socio-demographic characteristics |  |  |  |  |
| - age (mean; SD) | 51.7 (17.4) | 51.5 (16.1) | 50.9 (16.3) | 52.1 (16.0)* |
| - % woman | 64.2 | 70.5* | 70.5 | 70.8 |
| - % college/university | 16.2 | 15.3 | 14.1 | 15.7 |
| - % non-western | 8.1 | 3.7* | 5.6 | 2.4* |
| - % private insurance | 25.0 | 23.6 | 22.6 | 24.3 |
| - % living together | 78.6 | 79.7 | 79.5 | 78.9 |
| - % with job/study | 41.7 | 38.4 | 37.7 | 37.9 |
|  |  |  |  |  |
| **Use of medication** |  |  |  |  |
| Antidepressants |  |  |  |  |
| - % users of SSRIs | 42.3 | 58.7* | 65.5 | 55.9** |
| - % users of TCAs | 46.0 | 28.9* | 22.7 | 30.9** |
| - % users of other antidepressants | 11.7 | 12.4 | 11.7 | 13.2 |
| *Complex regime* |  |  |  |  |
| number of other ATCs (mean;sd) | 6.9 (5.8) | 6.0 (5.2)* | 5.6 (4.9) | 6.1 (5.2)** |
|  |  |  |  |  |
| Health & morbidity in general practice |  |  |  |  |
| *Self-reported health* |  |  |  |  |
| % excellent/good | 50.4 | 50.2 | 52.2 | 49.3 |
| *Diagnoses for which GP is consulted (% of patients)* |  |  |  |  |
| Depression (P03/P76) | 32.4 | 46.1* | 47.4 | 46.7 |
| Anxiety (P01/P74) | 14.4 | 18.3* | 17.2 | 19.0 |
| Neurasthenia (P78) | 6.5 | 3.0** | 2.6 | 3.2 |
| Other diagnoses in P-chapter (P01-P99) | 26.4 | 22.2* | 22.7 | 21.4 |
| *GP consultation for chronic diseases and overall contact* |  |  |  |  |
| Number of other chronic complaints (mean, SD) | 1.3 (1.5) | 1.1 (1.3)* | 1.1 (1.3) | 1.1 (1.3) |
| Number of contacts with GP (mean; SD) | 11.8 (10.2) | 11.5 (10.5) | 10.8 (9.9) | 11.9 (10.9)* |
| Total (%) | 11.4% | 88.6% | 24.6 | 75.4 |
| Number of patients (N) | 556 | 4,321 | 928 | 2,849 |

* P < 0.05
