# Supplementary material for: Patient risk profiles and practice variation in nonadherence to antidepressants, antihypertensives and oral hypoglycemics
Source: BMC Health Serv Res. 2007 Apr 10;7:51. doi: 10.1186/1472-6963-7-51 (PMC1855317; doi:10.1186/1472-6963-7-51)
Supplement: Additional file 2 — Appendix 2: Bivariate analyses antihypertensives. The table represents the results of the bivariate analyses for antihypertensives. [file 1472-6963-7-51-S2.doc]

# Appendix 2: Bivariate analyses antihypertensives

**Bivariate analyses on differences between early dropouts and continuers and on differences between seriously nonadherent patients (refill adherence)**

|  | **Early dropout** | | **Refill adherence** | |
| --- | --- | --- | --- | --- |
|  | Early dropout | Continuer | Nonadherent | Adherent |
| Socio-demographic characteristics |  |  |  |  |
| - age (mean; SD) | 51.1 (18.9) | 64.5 (13.4)* | 65.4 (14.3) | 64.8 (13,1) |
| - % woman | 66.2 | 57.5* | 61.4 | 56.9** |
| - % college/university | 16.0 | 10.5* | 10.7 | 10.2 |
| - % non-western | 5.9 | 2.4* | 97.7 | 98.0 |
| - % private insurance | 29.2 | 26.8 | 27.5 | 26.7 |
| - % living together | 84.0 | 76.4* | 72.7 | 76.9* |
| - % with job/study | 46.1 | 22.3* | 21.1 | 21.6 |
|  |  |  |  |  |
| **Medication** |  |  |  |  |
| Antihypertensives |  |  |  |  |
| - % users of beta blockers | 52.6 | 39.9* | 34.8 | 40.4* |
| - % users of diuretics | 34.3 | 32.9 | 48.9 | 30.3* |
| - % users of ace-inhibitors/A2 antagonists | 4.3 | 16.5* | 10.5 | 17.8* |
| - % users of other antihypertensives | 8.6 | 10.6* | 5.8 | 11.4* |
| *Complex regime* |  |  |  |  |
| number of other ATCs (mean;sd) | 5.6 (4.7) | 6.5 (4.9)* | 6.8 (5.3) | 6.6 (4.9) |
|  |  |  |  |  |
| Health & morbidity in general practice |  |  |  |  |
| *Self-reported health* |  |  |  |  |
| % excellent/good | 68.1 | 54.5* | 52.0 | 54.5* |
| *Diagnoses for which GP is consulted (% of patients)* |  |  |  |  |
| Diabetes (T90) | 4.9 | 14.8* | 13.0 | 15.6* |
| Hypertension (K85-K87) | 12.7 | 54.0* | 50.3 | 56.4* |
| Other diagnoses in K-chapter (from K70-K99) | 20.3 | 29.8* | 31.7 | 29.5* |
| Hypercholesterolemia (T93) | 2.8 | 8.5* | 7.5 | 8.9* |
| *GP consultation for chronic diseases and overall contact* |  |  |  |  |
| Number of other chronic complaints (mean, SD) | 0.9 (1.1) | 0.8 (1.0)* | 0.9 (1.1) | 0.8 (1.0)* |
| Number of contacts with GP (mean; SD) | 9.2 (7.6) | 10.2 (8.9)* | 11.0 (9.7) | 10.1 (8.9)* |
| Total (%) | 5.7% | 94.3% | 11.6 | 88.4 |
| Number of patients (N) | 813 | 13,406 | 1,405 | 10,705 |

* P < 0.05
